# The Yield of String Sign in Differentiating Mucinous from Non-Mucinous Pancreatic Cysts: A Retrospective Cross-Sectional Study

**DOI:** 10.3390/medicina57070716

**Published:** 2021-07-15

**Authors:** Wisam Sbeit, Anas Kadah, Amir Shahin, Tawfik Khoury

**Affiliations:** 1 Department of Gastroenterology, Galilee Medical Center, Nahariya 22100, Israel; wisams@gmc.gov.il (W.S.); anask@gmc.gov.il (A.K.); amirs3@gmc.gov.il (A.S.); 2 Faculty of Medicine in the Galilee, Bar-Ilan University, Safed 1311502, Israel

**Keywords:** cysts, pancreas, string sign, yield, EUS

## Abstract

*Background and Objectives*: The diagnosis of pancreatic cysts is mostly based on a combination of morphological appearance and fluid analysis of amylase and carcinoembryonic antigen (CEA). We aimed to assess the capability of the string sign in differentiating mucinous from non-mucinous pancreatic cysts. *Materials and Methods:* All patients who were referred for endoscopic ultrasound (EUS) for pancreatic cysts assessment from 2015 to 2020 were retrospectively analyzed. *Results:* Our cohort consisted of 112 patients. Of them, 92 patients (82.1%) had mucinous cystic neoplasms (group A) and 20 patients (17.9%) had non-mucinous cystic neoplasms (group B). The average age in groups A and B was 71.3 and 60.4 years, respectively. String sign was positive in 47 patients (51.1%) and negative in 21 patients (22.8%) in group A, while in group B, string sign was negative in 19 patients (95%). String sign showed significant correlation with the diagnosis of mucinous cystic neoplasms (OR 64.2, 95% CI 8.1–508.6, *p* = 0.0001). Cytology confirmed mucinous cystic neoplasms that included 32 patients; the sensitivity, specificity, positive predictive value (PPV), and negative predictive value (NPV) of string sign for mucinous cystic neoplasms were high, reaching 93.8%, 85.7%, 96.8%, and 75%, respectively, with an excellent accuracy rate of 92.3%. *Conclusions:* The string sign is highly accurate for predicting pancreatic mucinous cystic neoplasms, and should be used as an important aid for improving diagnostic accuracy.

## 1. Introduction

The diagnosis of pancreatic cysts has increased in recent years due to the wide use of abdominal radiology. Approximately 2.5% of pancreatic cysts are diagnosed incidentally [1,2].

Basically, pancreatic cysts can be either mucinous or non-mucinous. Endoscopic ultrasound (EUS) is currently the preferred method for diagnosing pancreatic cysts because it enables fluid acquisition. EUS showed improved morphological diagnosis of pancreatic cysts [3,4], however, diagnostic accuracy is still limited and has been shown to approach almost 50% in a large multicenter study [5]. Thus, other methods are used to optimize the yield of pancreatic cysts diagnosis, including biochemical analysis of the cystic fluid [6]. However, the yield of biochemical cystic fluid analysis is still limited. Previous meta-analysis reached the conclusion that EUS-fine needle aspiration (FNA) has a low sensitivity, but high specificity [7]. Recently, some studies have reported a high diagnostic yield for pancreatic cystic lesions with worrisome features by using through-the-needle microforceps biopsy of the pancreatic cystic wall [8,9]. Moreover, Mohan et al. have reported an intra-cystic cut-off glucose level of 50 mg/dL as an excellent diagnostic test in differentiating mucinous from non-mucinous pancreatic cysts [10].

Another immediate on-site method is the string sign which is done by measuring the maximal length of mucus without being disrupted when stretched between the examiner fingers [11]. Most previous studies have focused on both EUS morphology and FNA biochemical analysis for the diagnosis of pancreatic cystic neoplasm, however there is a scarcity of research regarding the yield of string sign in this setting. Therefore, we aimed to explore the diagnostic yield of string sign in differentiating mucinous from non-mucinous pancreatic cysts.

## 2. Materials and Methods

This is a retrospective single center study conducted at Galilee Medical Center. Inclusion criteria included patients 18 years of age or older, who underwent EUS examinations for further assessment of pancreatic cysts between 2015 to 2020. Patients were excluded if they had solid pancreatic lesions on EUS or had not undergone FNA due to contraindications. Extracted data included demographics, EUS diagnosis as diagnosed by pre-defined morphological diagnosis and biochemical cyst fluid analysis results including Carcinoembryonic antigen (CEA), amylase and cytology and string sign result. All FNAs punctures were obtained via 22 Gauge needle (COOK MEDICAL, echo tip ultra, Ireland). The study was approved by the local institutional board. Written informed consent was waived due to the retrospective non-interventional study design.

### 2.1. Morphologic Sonographic Characterization of Pancreatic Cysts

Our study cohort involved 4 types of pancreatic cysts including: (1) IPMNs—main-duct IPMNs are defined by non-obstructive pancreatic duct dilatation, while the branch duct type is defined by cystic dilation of side branches connected to the main pancreatic duct, whereas the mixed type is defined by a combination of main and branch ducts [12]. (2) MCN—defined by multiple or single cyst not connected to the main pancreatic duct, usually less than 6 cm, affecting mainly females, and occurring in the pancreatic body and tail [12,13]. (3) SCA—defined by a focal cystic lesion occurring in all pancreatic parts, and may be oligo-cystic or microcystic with pathognomonic central calcification in up to 20% of cases [14], and (4) PC—defined by single peri-pancreatic single cyst appearing mainly following an episode of acute pancreatitis or after traumatic abdominal injury [15].

### 2.2. Biochemical Analysis

Biochemical analysis (Amylase and CEA levels) was examined in the pancreatic cystic fluid. To date, the diagnostic accuracy of CEA and amylase levels are limited given their association with variable sensitivity and specificity making interpretation difficult [5,16]. The professional guideline set by the American society of gastrointestinal endoscopy (ASGE) has settled on a cut-off level of CEA for mucinous cystic neoplasms of >192 ng/mL and for serous cysts of <5 ng/mL [15]. A similar guideline was released by World Gastroenterology, which reported the same CEA cutoff level for mucinous cystic neoplasms of >192 ng/mL (sensitivity 73%, specificity 84%) and for serous cysts of <5 ng/mL (sensitivity 100% and specificity 86%) (WGO Global Guideline for Pancreatic Cystic Lesions 2019). Therefore, we used the globally used cutoff CEA of >192 ng/mL to assess the correlation between the morphological diagnosis of mucinous cystic neoplasms with the biochemical cyst fluid analysis diagnosis.

### 2.3. String Sign Definition

String sign was reported in all EUS examinations performed for cystic pancreatic lesions during the study period. It was performed by placing a drop of cystic fluid between the examiner’s thumb and index finger, and slowly separating the fingers until a string is produced to its maximal length, then measured by a ruler between the fingers. String sign was considered positive if a string of ≥1 cm was produced and lasted for ≥1 s [17].

### 2.4. Diagnosis of Pancreatic Cysts

The diagnosis of pancreatic cysts in our cohort was based on a combination of endosonographic morphological appearance coupled with biochemical cyst analysis, and cytology when informative.

### 2.5. Statistical Analysis

Categorial variables were analyzed by Fisher’s exact tests and reported as percentages, while continuous variables were analyzed by t-test and reported as mean ± SD. The diagnostic performance of string sign for mucinous pancreatic cysts was reported by sensitivity, specificity, positive predictive value, and negative predictive value. The cut-off point for cyst CEA level was determined using receiver operating characteristics (ROC) analysis with the reported Youden index (J). We determined the diagnostic accuracy of the cut-off point generated using sensitivity and specificity, positive and negative predictive values. Statistical significance was set at *p* < 0.05. All analyses were performed using statistical analysis software (SAS vs. 9.4 Copyright (c) 2016 by SAS Institute Inc., Cary, NC, USA).

## 3. Results

### 3.1. Demographics, Chemical and Morphological Sonographic Characteristics

The study cohort consisted of 112 patients who underwent EUS-FNA for pancreatic cysts. Among them, 92 patients (82.1%) were diagnosed with mucinous cysts (group A), while 20 patients (17.9%) were diagnosed with non-mucinous cysts (group B). The average age was 71.3 ± 11.2 in group A and 60.4 ± 14.9 in group B. Thirty-eight patients (41.3%) in group A were males, compared to 10 patients (50%) in group B. The mean cyst amylase and CEA levels were significantly higher in group A compared to group B (45,032 unit/lit vs. 5467 unit/lit and 886 ng/mL vs. 129 ng/mL, respectively). Table 1 demonstrates the demographics, and endoscopic and cyst fluid analysis findings.

### 3.2. The Rate of Positive String Sign in Our Study Cohort

String sign was positive in 71 patients (77.2%) and negative in 21 patients (22.8%) in group A. String sign was positive in only 1 patient (5%) in group A. The only patient in group B that had a positive string sign was diagnosed with PC. Sensitivity, specificity, positive predictive value (PPV), and negative predictive value (NPV) for positive string sign in predicting mucinous pancreatic cystic neoplasms in the entire cohort were 77.2%, 95%, 98.6%, and 47.5%, respectively, with accuracy of 80.4% (Table 2). Notably, positive string sign was significantly correlated with the diagnosis of mucinous pancreatic cysts (OR 64.2, 95% CI 8.1–508.6, *p* = 0.0001).

### 3.3. Correlation of Positive String Sign with Cut-Off Level of Cyst Amylase and CEA Levels

The mean cyst amylase levels in both the positive and negative string sign groups were similar (43671 ± 110,505 unit/lit vs. 39,303 ± 76,505 unit/lit, respectively, *p* = 0.4), however, the cyst level of CEA was significantly higher among the positive string sign group (1283 ± 2536 ng/mL) vs. the negative string sign group (449 ± 1216 ng/mL) (*p* = 0.03). Notably, sensitivity, specificity, PPV, and NPV of string sign for the CEA cut-off level of CEA of >192 ng/mL for differentiating mucinous from non-mucinous pancreatic cysts according to the international guidelines in the entire cohort were 74.4%, 94.4%, 96.7% and 63% (Table 3).

### 3.4. Positive Cytology Group Analysis and It’s Correlation with String Sign

**Figure 1:**
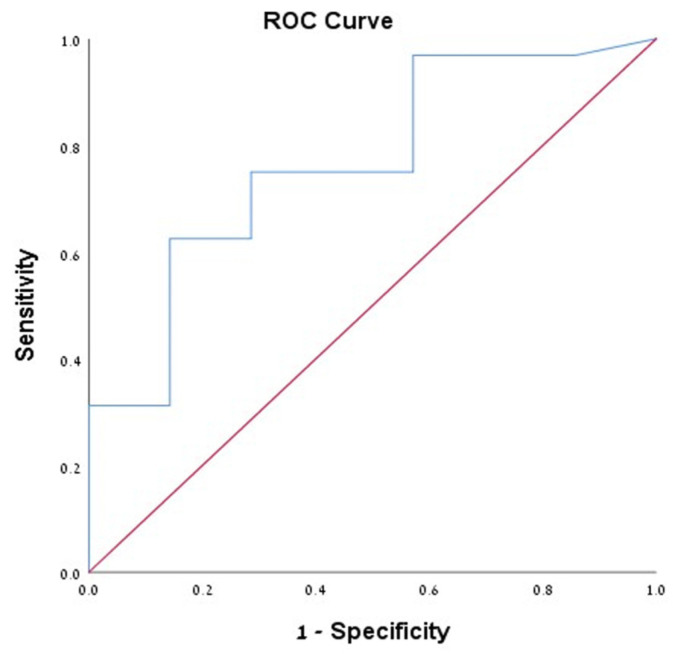
Demonstrating the ROC of cyst level of CEA among patients with cytologically confirmed mucinous cystic neoplasms.

Overall, 39 patients of the entire cohort (34.8%) had positive cytology results. Among them, 32 patients (82%) had both morphologically and cytologically confirmed diagnosis of mucinous cysts. The sensitivity, specificity, PPV, and NPV of string sign with mucinous cystic neoplasms in the cytology positive group were high and reached 93.8%, 85.7%, 96.8%, and 75%, respectively, with accuracy of 92.3% (Table 2), supporting its excellent diagnostic accuracy in predicting mucinous cysts. Moreover, we performed ROC analysis for CEA in patients with both ultrasonographic morphological and cytological diagnosis of mucinous cysts. The ROC of CEA was 0.766 (95% CI 0.575–0.956) (Figure 1); for CEA values according to the Youden index of 25.5 ng/mL or more, associated sensitivity, specificity, PPV, NPV, and accuracy of 81.9%, 92.3%, 98.3%, 48%, and 83.5%, respectively, were recorded, compared to 74.4%, 94.4%, 96.7%, 63%, and 80.7%, respectively, in the CEA > 192 group. Interestingly, in the cytologically positive group, the sensitivity, specificity, PPV, NPV, and accuracy of string sign for CEA cut-off level of CEA of >25.5 ng/mL were 92%, 80%, 95.8%, 66.7%, and 90%, respectively, while for CEA > 192 ng/mL patients, values were 92.3%, 83.3%, 92.3%, 83.3%, and 89.5%, respectively (Table 3), showing similar diagnostic accuracy between the two CEA cut-off levels.

## 4. Discussion

Accurate diagnosis of pancreatic cyst type has tremendous significance, since mucinous cysts are premalignant and require surveillance and ultimately surgical resection [18,19,20]. Unfortunately, the yield of cyst fluid cytology is low. De Jong, et al. have reported diagnostic EUS FNA cytology in about third of cases [21]. On the other hand, cyst fluid viscosity is higher in mucinous cysts [22], as reflected by the positive string sign.

Our study demonstrated the high performance of the string sign in differentiating mucinous from non-mucinous pancreatic cysts, as evidenced by its high diagnostic performance, and showed a significant correlation with the diagnosis of mucinous cystic neoplasms (OR 64.2, 95% CI 8.1–508.6, *p* = 0.0001). Moreover, the diagnostic performance improved in the positive cytology subgroup, supporting its excellent diagnostic accuracy in diagnosing mucinous cystic neoplasms. Notably, only 1 patient (5%) with a non-mucinous cyst had a positive string sign. In their study, Bick BL et al. showed a high specificity of string sign in diagnosing mucinous cysts, as it improved the diagnostic accuracy when added to cyst fluid analysis [17]. Another study by Leung KK et al. has reported that benign pancreatic cysts had a median string sign length of 0 mm compared to 3.5 mm in mucinous cysts, and they showed that string sign length of 1 mm reflects a higher fluid viscosity and increases the likelihood of identifying a mucinous cyst by 116% [11]. In our study, we obtained similar results regarding the predictive potential of string sign for the diagnosis of pancreatic cysts to those reported in the literature, thus supporting the role of the string sign as an important immediate tool in distinguishing cystic lesions.

Interestingly, in further sub-analysis, we showed that among the cytologically positive group, the sensitivity, specificity, PPV, NPV, and accuracy of string sign among patients of both groups of cut-off level of CEA > 25.5 ng/mL and cut off level of CEA > 192 ng/mL were very high and similar. These findings indicate the need for revision of this cut off value of CEA > 192 ng/mL. To date, the reported cut-off value for CEA by professional international societies was shown to have variable sensitivities and specificities as has been demonstrated by several studies. In their study that included 112 patients with surgically resected pancreatic cysts, Brugge WR et al. showed that a cutoff value of CEA > 192 ng/mL had a sensitivity and specificity of 73% and 83%, respectively, for diagnosing a pancreatic mucinous cyst [5]. A second study that recruited 198 surgically resected pancreatic cysts reported that a cutoff value of 109.9 ng/mL had a sensitivity and specificity of 81% and 98%, respectively, for diagnosing a mucinous cyst [23], further addressing the conflicting results regarding the diagnostic performance of CEA > 192 ng/mL for mucinous cysts.

Recently, molecular and liquid sample analysis has been reported to improve the diagnostic yield of mucinous pancreatic cysts with potentially high-grade lesions, however, the experience with these tests is still limited, as they are not easily available at every center, are time consuming, and require resources [24]. Whereas use of the string sign is a simple, bedside, cost-free test providing an immediate diagnosis.

The limitations of our study include the retrospective design and that it was conducted at a single center. An additional limitation is that we classified the pancreatic cysts according to the sonographic morphological diagnosis, however, due to the fact that all patients who received morphological diagnosis of mucinous cysts were confirmed by positive cytology for mucinous cysts, which reflects the accuracy of the sonographic morphological criteria used, and thus can be generalized for the entire study cohort.

## 5. Conclusions

Our study showed that string sign was associated with excellent diagnostic performance for pancreatic mucinous cystic neoplasms, even more than CEA level. The on-site string sign test is a Rapid On-Site Evaluation (ROSE) test of the fluid viscosity, resembling the rapid on-site evaluation of cytology from a solid pancreatic lesion, which needs only a small amount of fluid from the same FNA, and provides immediate results complementary to the morphological characteristics, and biochemical and cytological analysis of the cyst. This test seems to contribute to the above-mentioned suboptimal tests in differentiating between mucinous and non-mucinous pancreatic cysts with its implications for the management of pancreatic cysts. Further multicenter studies are needed to evaluate the precise role of the string sign ROSE as a complementary test to the available armamentarium in diagnosing pancreatic cystic lesions.

## Figures and Tables

**Table 1:** Demographics, chemical, and morphological sonographic characteristics.

	Group A (Mucinous Cystic Neoplasms)	Group B (Non-Mucinous Cysts)
Number of patients	92	20
Age (mean ± SD)	71.3 ± 11.2	60.4 ± 14.9
Gender, *N*(%)		
Male	38 (41.3)	10 (50)
Female	54 (58.7)	10 (50)
Pancreatic Cysts types, *N*(%)		
IPMN	81 (88)	0
MCN	11 (12)	0
SCA	0	15 (75)
PC	0	5 (25)
Amylase, unit/lit (mean ± SD)	45,032 ± 98,478	5467 ± 11,004
CEA, ng/mL (mean ± SD)	886 ± 1999	129 ± 343
Maximal cyst size (mm)	22.2 ± 14.4	31.2 ± 22.8
Positive string sign, *N*(%)	47 (51.1)	1 (5)

CEA: carcinoembryonic antigen.

**Table 2:** Diagnostic performance of the string sign.

	Sensitivity	Specificity	PPV	NPV	Diagnostic Accuracy
String sign yield in all cohort with mucinous cysts	77.2%	95%	98.6%	47.5%	80.4%
String sign yield in cytologically confirmed mucinous cysts	93.8%	85.7%	96.8%	75%	92.3%

**Table 3:** Diagnostic performance of the string sign for two CEA cut-off levels in the entire cohort and in the cytology positive group.

	Sensitivity	Specificity	PPV	NPV	Diagnostic Accuracy
CEA of > 192 ng/mL					
All cohort	74.4%	94.4%	96.7%	63%	80.7%
Cytologically positive cohort	92.3%	83.3%	92.3%	83.3%	89.5%
CEA of > 25.5 ng/mL					
All cohort	81.9%	92.3%	98.3%	48%	83.5%
Cytologically positive cohort	92%	80%	95.8%	66.7%	90%

## Data Availability

The data of the study are present at the Gastroenterology department at Galilee Medical Center. The data will be available upon reasonable request.

## Abbreviations

IPMN: Intraductal papillary mucinous neoplasm; MCN: mucinous cystic neoplasms; SCA: serous cystic adenoma; CT: computed tomography; EUS: endoscopic ultrasound; FNA: fine needle aspiration; CEA: Carcinoembryonic antigen; PC: pseudocyst; ASGE: American society of gastrointestinal endoscopy; OR: odds ratio; ROC: receiver operating characteristics; PPV: positive predictive value; NPV: negative predictive value.

## References

[1] De Jong K., Nio C.Y., Mearadji B., Phoa S.S., Engelbrecht M.R., Dijkgraaf M.G., Bruno M.J., Fockens P. (2012). Disappointing interobserver agreement among radiologists for a classifying diagnosis of pancreatic cysts using magnetic resonance imaging. Pancreas.

[2] Laffan T.A., Horton K.M., Klein A.P., Berlanstein B., Siegelman S.S., Kawamoto S., Johnson P.T., Fishman E.K., Hruban R.H. (2008). Prevalence of unsuspected pancreatic cysts on MDCT. Am. J. Roentgenol..

[3] Ahmad N.A., Kochman M.L., Lewis J.D., Ginsberg G.G. (2001). Can EUS alone differentiate between malignant and benign cystic lesions of the pancreas?. Am. J. Gastroenterol..

[4] Palazzo L., Roseau G., Gayet B., Vilgrain V., Belghiti J., Fékété F., Paolaggi J.-A. (1993). Endoscopic ultrasonography in the diagnosis and staging of pancreatic adenocarcinoma. Endoscopy.

[5] Brugge W.R., Lewandrowski K., Lee-Lewandrowski E., Centeno B.A., Szydlo T., Regan S., del Castillo C.F., Warshaw A.L. (2004). Diagnosis of pancreatic cystic neoplasms: A report of the cooperative pancreatic cyst study. Gastroenterology.

[6] Khashab M.A., Kim K., Lennon A.M., Shin E.J., Tignor A.S., Amateau S.K., Singh V.K., Wolfgang C.L., Hruban R.H., Canto M.I. (2013). Should we do EUS/FNA on patients with pancreatic cysts? The incremental diagnostic yield of EUS over CT/MRI for prediction of cystic neoplasms. Pancreas.

[7] Thornton G., McPhail M., Nayagam A.S., Hewitt M., Vlavianos P., Monahan K. (2013). Endoscopic ultrasound guided fine needle aspiration for the diagnosis of pancreatic cystic neoplasms: A meta-analysis. Pancreatology.

[8] Larghi A., Manfrin E., Fabbri C., Crinò S.F., Correale L., Chiarello G., Barresi L., van Velthuysen M.-L., Poley J.W., Rahal D. (2019). Interobserver agreement among expert pathologists on through-the-needle microforceps biopsy samples for evaluation of pancreatic cystic lesions. Gastrointest. Endosc..

[9] Crinò S.F., Bernardoni L., Gabbrielli A., Capelli P., Salvia R., Rusev B.C., Scarpa A., Manfrin E. (2018). Beyond Pancreatic Cyst Epithelium: Evidence of Ovarian-Like Stroma in EUS-Guided Through-the-Needle Micro-Forceps Biopsy Specimens. Am. J. Gastroenterol..

[10] Khan S.R., Mohan B.P., Madhu D., Chandan S., Kassab L., Ponnada S., Facciorusso A., Crinò S.F., Barresi L., Adler D.G. (2021). ID: 3520672 Intracystic Glucose Levels in Differentiating Mucinous from Non-Mucinous Pancreatic Cysts: A Systematic Review and Meta-Analysis. Gastrointest. Endosc..

[11] Leung K.K., Ross W.A., Evans D., Fleming J., Lin E., Tamm E.P., Lee J.H. (2009). Pancreatic cystic neoplasm: The role of cyst morphology, cyst fluid analysis, and expectant management. Ann. Surg. Oncol..

[12] Das A., Ngamruengphong S., Nagendra S., Chak A. (2009). Asymptomatic pancreatic cystic neoplasm: A cost-effectiveness analysis of different strategies of management. Gastrointest. Endosc..

[13] Brugge W.R. (2015). Diagnosis and management of cystic lesions of the pancreas. J. Gastrointest. Oncol..

[14] Kubo H., Nakamura K., Itaba S., Yoshinaga S., Kinukawa N., Sadamoto Y., Ito T., Yonemasu H., Takayanagi R. (2009). Differential diagnosis of cystic tumors of the pancreas by endoscopic ultrasonography. Endoscopy.

[15] Muthusamy V.R., Chandrasekhara V., Acosta R.D., Bruining D.H., Chathadi K.V., Eloubeidi M.A., Faulx A.L., Fonkalsrud L., Gurudu S.R., ASGE Standards of Practice Committee (2016). The role of endoscopy in the diagnosis and treatment of cystic pancreatic neoplasms. Gastrointest. Endosc..

[16] Frossard J.L., Amouyal P., Amouyal G., Palazzo L., Amaris J., Soldan M., Giostra E., Spahr L., Hadengue A., Fabre M. (2003). Performance of endosonography-guided fine needle aspiration and biopsy in the diagnosis of pancreatic cystic lesions. Am. J. Gastroenterol..

[17] Bick B.L., Enders F.T., Levy M.J., Zhang L., Henry M.R., Abu Dayyeh B.K., Chari S.T., Clain J.E., Farnell M.B., Gleeson F.C. (2015). The string sign for diagnosis of mucinous pancreatic cysts. Endoscopy.

[18] Ryu J.K., Woo S.M., Hwang J.-H., Jeong J.B., Yoon Y.B., Park I.A., Han J.K., Kim Y.-T. (2004). Cyst fluid analysis for the differential diagnosis of pancreatic cysts. Diagn. Cytopathol..

[19] Goh B.K., Tan Y.-M., Cheow P.-C., Chung Y.-F.A., Chow P., Wong W.-K., Ooi L.L. (2006). Cystic lesions of the pancreas: An appraisal of an aggressive resectional policy adopted at a single institution during 15 years. Am. J. Surg..

[20] Moparty B., Logroño R., Nealon W.H., Waxman I., Raju G.S., Pasricha P.J., Bhutani M.S. (2007). The role of endoscopic ultrasound and endoscopic ultrasound-guided fine-needle aspiration in distinguishing pancreatic cystic lesions. Diagn. Cytopathol..

[21] De Jong K., Poley J.-W., Van Hooft J.E., Visser M., Bruno M.J., Fockens P. (2011). Endoscopic ultrasound-guided fine-needle aspiration of pancreatic cystic lesions provides inadequate material for cytology and laboratory analysis: Initial results from a prospective study. Endoscopy.

[22] Lewandrowski K.B., Southern J.F., Pins M.R., Compton C.C., Warshaw A.L. (1993). Cyst Fluid Analysis in the Differential Diagnosis of Pancreatic Cysts A Comparison of Pseudocysts, Serous Cystadenomas, Mucinous Cystic Neoplasms, and Mucinous Cystadenocarcinoma. Ann. Surg..

[23] Cizginer S., Turner B.G., Bilge A.R., Karaca C., Pitman M.B., Brugge W.R. (2011). Cyst fluid carcinoembryonic antigen is an accurate diagnostic marker of pancreatic mucinous cysts. Pancreas.

[24] Haeberle L., Schramm M., Goering W., Frohn L., Driescher C., Hartwig W., Preissinger-Heinzel H.-K., Beyna T., Neuhaus H., Fuchs K. (2021). Molecular analysis of cyst fluids improves the diagnostic accuracy of pre-operative assessment of pancreatic cystic lesions. Sci. Rep..

